# Supplementary material for: TECRL deficiency results in aberrant mitochondrial function in cardiomyocytes
Source: Commun Biol. 2022 May 16;5:470. doi: 10.1038/s42003-022-03414-9 (PMC9110732; doi:10.1038/s42003-022-03414-9)
Supplement: Supplementary file 2 — Supplementary Information [file 42003_2022_3414_MOESM2_ESM.pdf]

Figure legends

Fig S1. Tecrl deficiency did not induce cardiac dysfunction at four to five weeks of age. (a) Representative echocardiography images of WT and Tecrl KO mice at the age of four to five weeks. (b) Quantification of LVEF, LVFS, LVIDd, LVIDs, LVEDV, and LVESV in WT and Tecrl KO mice (four to five weeks) ($n = 6$). (c) Representative immunoblotting images of TECRL of the patient and his parents. Values are mean ± SE. $P < 0.05$ was considered significant.

Fig S2. Tecrl deficiency increases reactive oxygen species production. (a, b) Representative images of the DHE staining and statistical charts. The WT and Tecrl KO mouse tissues were measured at the age of four to five weeks ($n = 6$). Values are mean ± SE. $P < 0.05$ was considered significant.

Fig S3. Visualization of differentially regulated proteins in mitochondria isolated from the WT and Tecrl KO mice hearts. (a) Volcano plots of differentially regulated proteins in cardiac mitochondria, those achieving $P < 0.05$ and $|\text{fold-change}| > 1.3$ are highlighted. (b) KEGG enrichment analysis of the differentially expressed proteins in WT and Tecrl KO mouse mitochondria.

Fig S4. Overexpression of TECRL can induce mitochondrial respiration in H9C2 cells. (a-d) The relative mRNA levels of NRF2, FAS, MFN2, and TECRL in H9C2 cells after siTECRL treatment ($n = 6$). (e-g) Measure of OCR and respective quantitative analysis in H9C2 cells ($n = 12$), FCCP, trifluoromethoxy carbonyl cyanide phenylhydrazone. Values are mean ± SE. $P < 0.05$ was considered significant.

Fig S5. TECRL effects on NRF2, MFN2, and FAS in H9C2. (a-c) Representative immunoblotting images and quantification of the expression of NRF2, MFN2, FAS, and TECRL in H9C2 following TECRL knockdown using siRNA ($n=8$). Values are mean ± SE. $P < 0.05$ was considered significant.

Fig S6. Construction of the Tecrl KO mice. (a) The location of the primers. (b) The sequence of the primers.

Table information

Table S1. The relative protein expression of WT and Tecrl KO mouse hearts through mass spectrometry.

Table S2. The relative differential expression of genes in WT and Tecrl KO mouse hearts through RNA-sequencing.

Table S3. A total of 306 (16.2%) cardiac proteins were differentially expressed in Tecrl KO mice relative to WT, observed through mitochondrial proteomics detection.

Table S4. Baseline of contraction of TECRL knockdown hiPSC-CMs versus baseline.

Table S5. All the expression of proteins after TECRL overexpression in the hiPSC-CMs through mass spectrometry.

Table S6. All the expression of proteins after co-immunoprecipitation overexpressed by TECRL in the hiPSC-CMs through mass spectrometry.

Video S1. The representative video of cardiomyocytes (hiPSC-CMs) beating spontaneously.

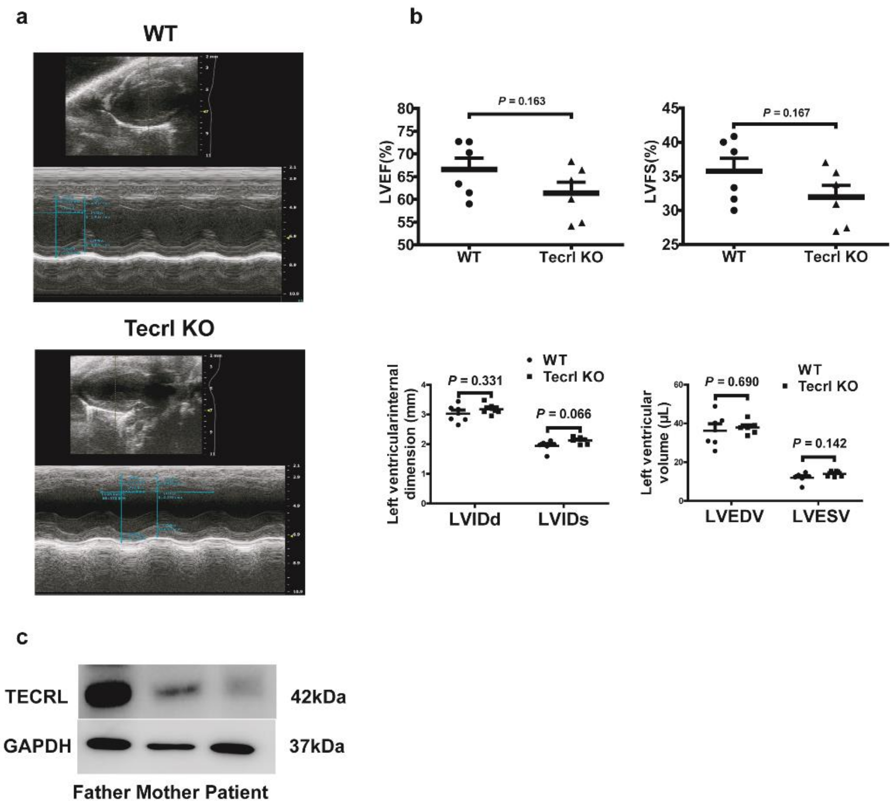

Fig S1. Tecrl deficiency did not induce cardiac dysfunction at four to five weeks of age. (a) Representative echocardiography images of WT and Tecrl KO mice at the age of four to five weeks. (b) Quantification of LVEF, LVFS, LVIDd, LVIDs, LVEDV, and LVESV in WT and Tecrl KO mice (four to five weeks) (n = 6). (c) Representative immunoblotting images of TECRL of the patient and his parents. Values are mean ± SE. $P < 0.05$ was considered significant.

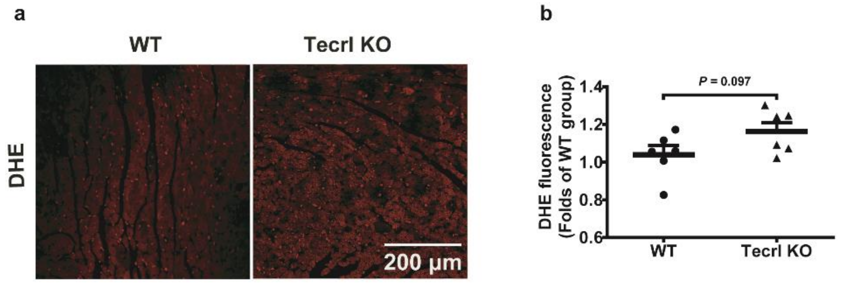

Fig S2. Tecrl deficiency increases reactive oxygen species production. (a, b) Representative images of the DHE staining and statistical charts. The WT and Tecrl KO mouse tissues were measured at the age of four to five weeks (n = 6). Values are mean ± SE. $P < 0.05$ was considered significant.

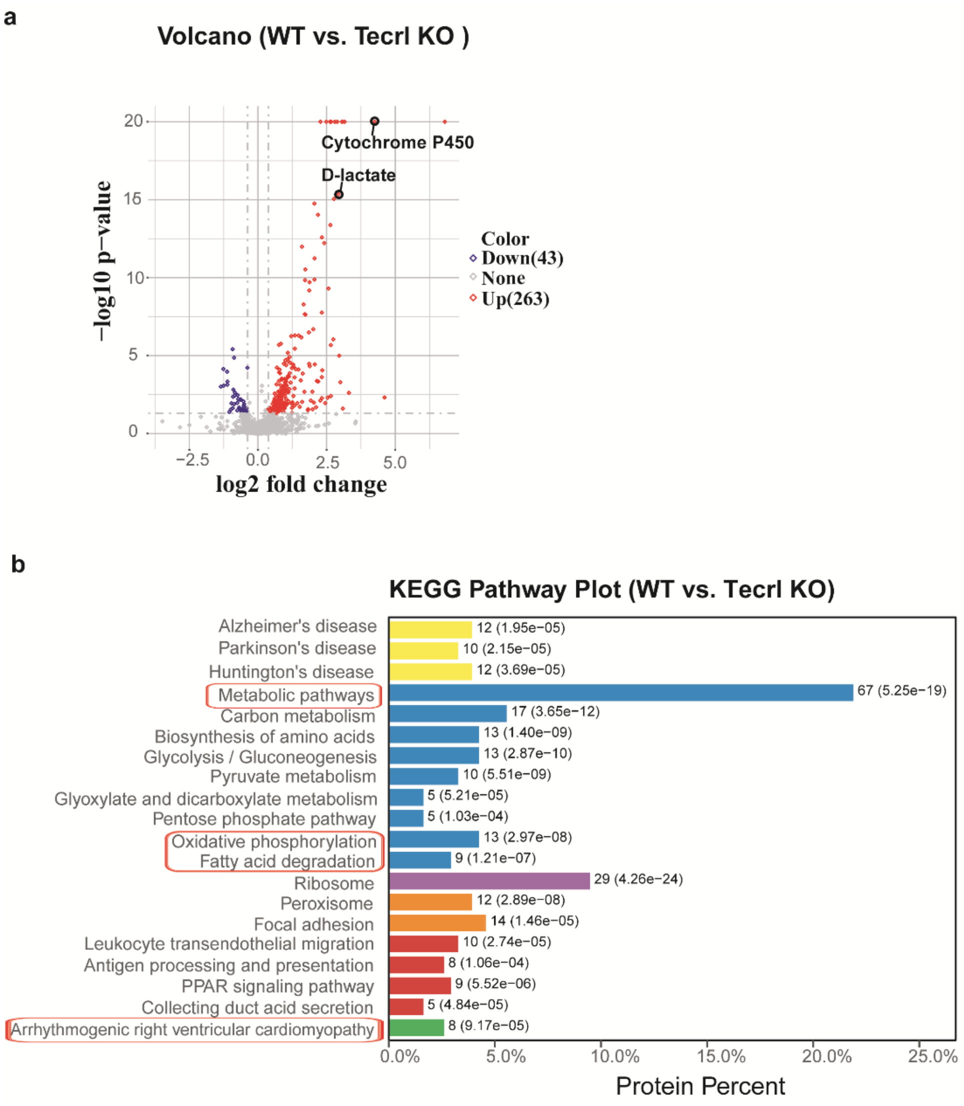

Fig S3. Visualization of differentially regulated proteins in mitochondria isolated from the WT and Tecrl KO mice hearts. (a) Volcano plots of differentially regulated proteins in cardiac mitochondria, those achieving $P < 0.05$ and |fold-change| > 1.3 are highlighted. (b) KEGG enrichment analysis of the differentially expressed proteins in WT and Tecrl KO mouse mitochondria.

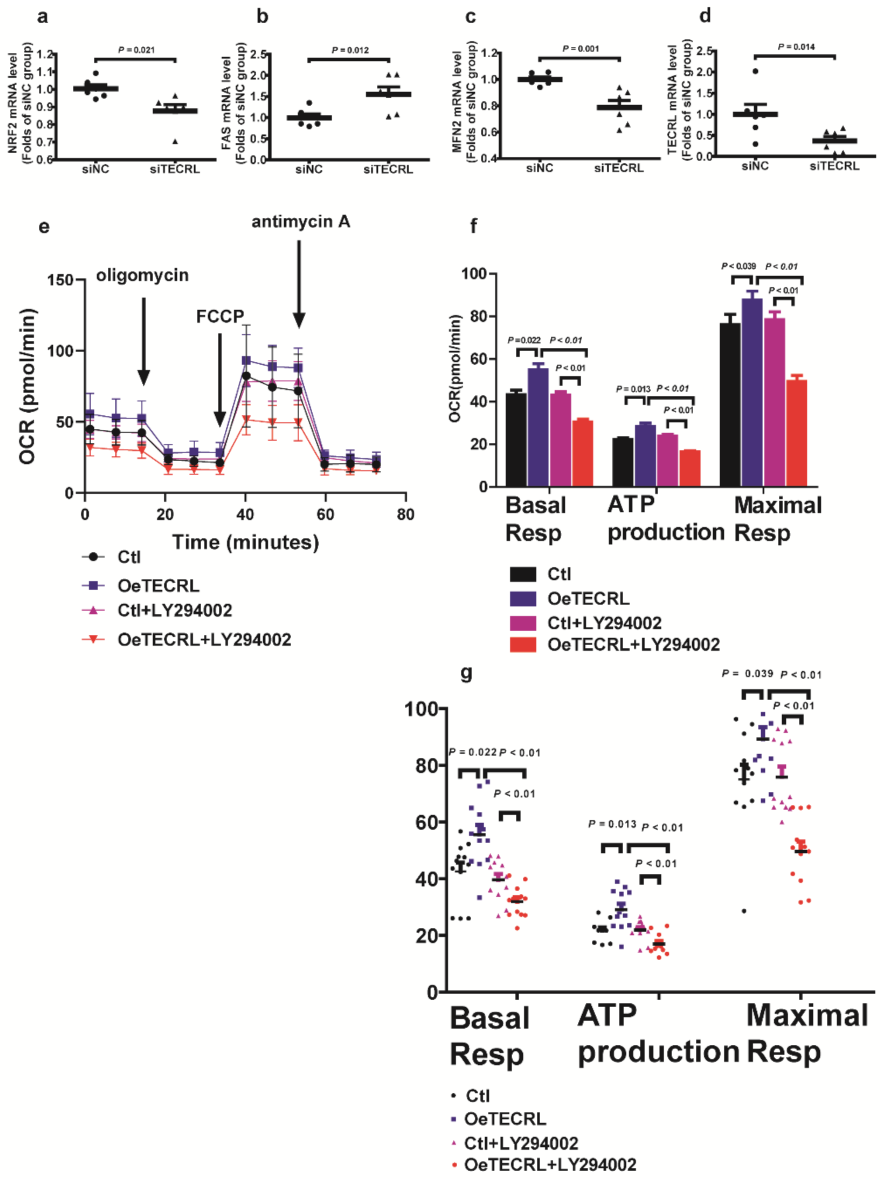

Fig S4. Overexpression of TECRL can induce mitochondrial respiration in H9C2 cells. (a-d) The relative mRNA levels of NRF2, FAS, MFN2, and TECRL in H9C2 cells after siTECRL treatment (n = 6). (e-g) Measure of OCR and respective quantitative analysis in H9C2 cells (n = 12), FCCP, trifluoromethoxy carbonyl cyanide phenylhydrazone. Values are mean ± SE. $P < 0.05$ was considered significant.

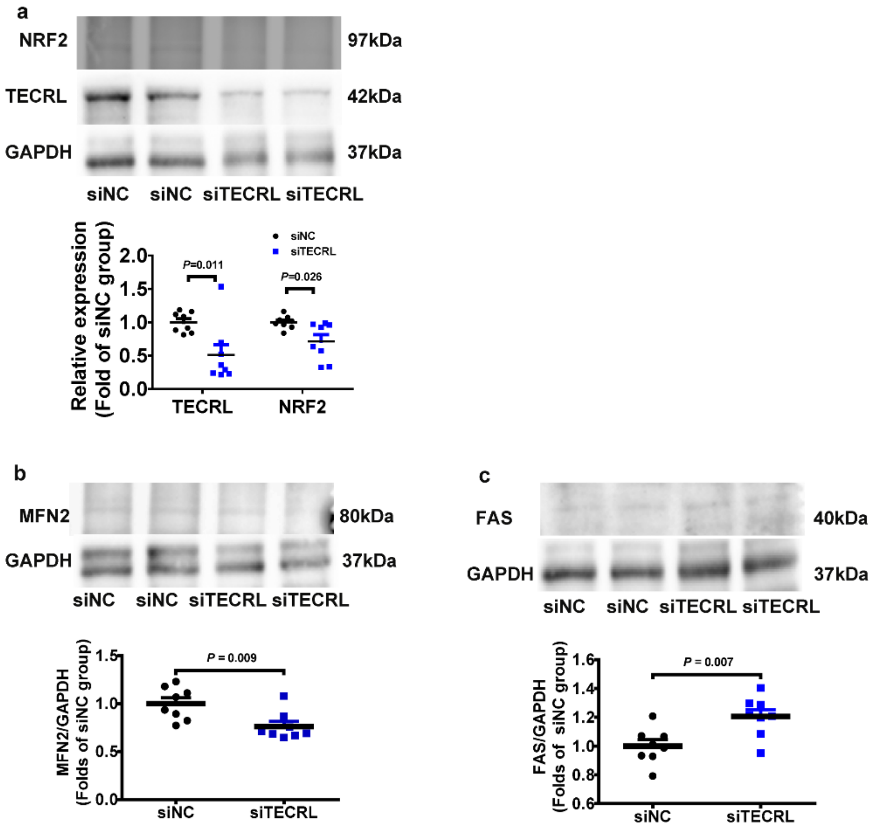

Fig S5. TECRL effects on NRF2, MFN2, and FAS in H9C2. (a-c) Representative immunoblotting images and quantification of the expression of NRF2, MFN2, FAS, and TECRL in H9C2 following TECRL knockdown using siRNA (n=8). Values are mean ± SE. $P < 0.05$ was considered significant.

a

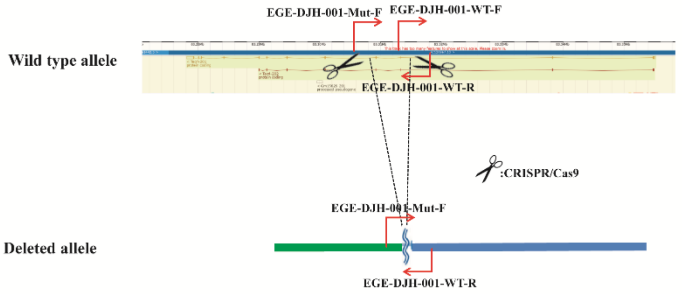

b

Primer	Sequence (5'-3')	Tm (℃)	Product size (bp)
EGE-DJH-001-WT-F	TGTATTGCACAGTTTTGGGCTCATGG	61	WT:455
EGE-DJH-001-WT-R	TGCACTTGAATGGAAAAAGACTGGA	58	
EGE-DJH-001-Mut-F	CATAGGGACTCGATTGTTGTCCGTG	60	WT:6033
EGE-DJH-001-WT-R	TGCACTTGAATGGAAAAAGACTGGA	58	Mut:537

Fig S6. Construction of the Tecrl KO mice. (a) The location of the primers. (b) The sequence of the primers.

Supplementary figure of uncropped blots

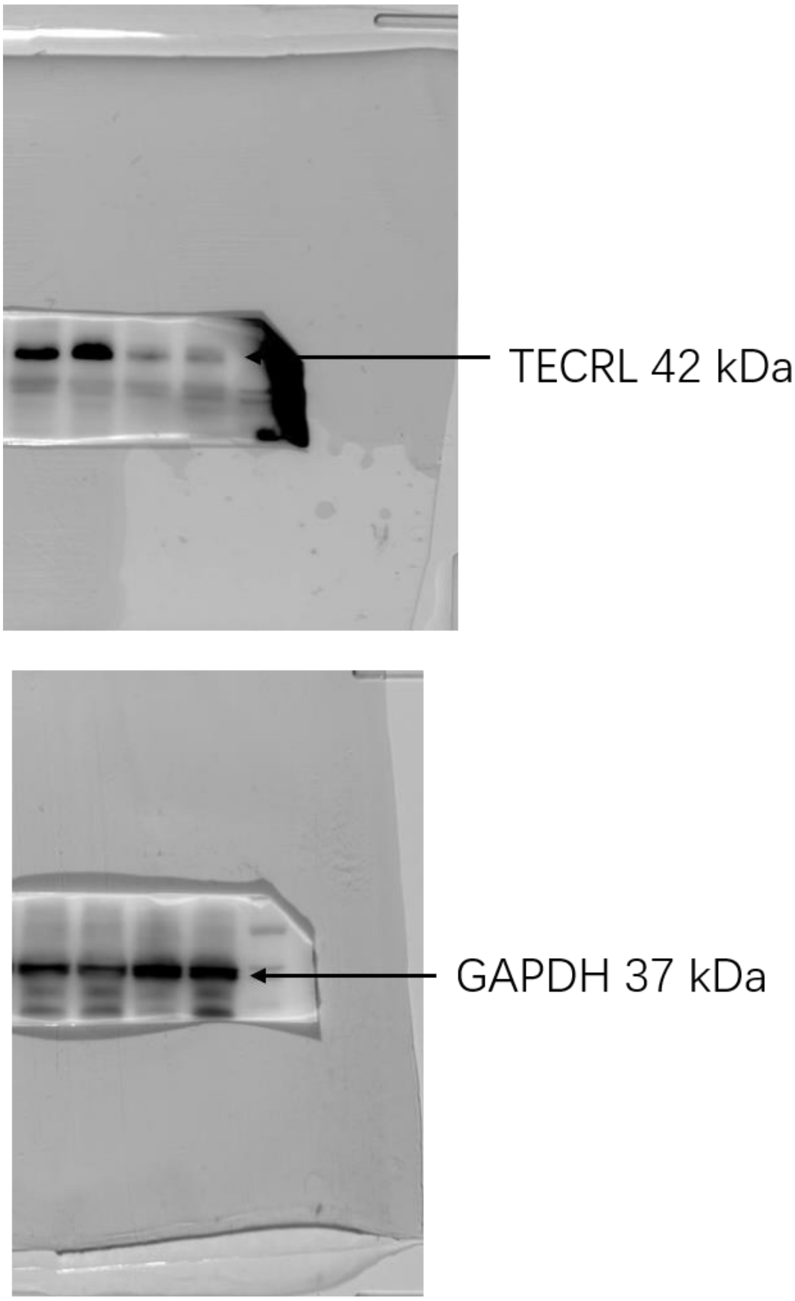

Fig S7. Uncropped blots of Fig 1e.

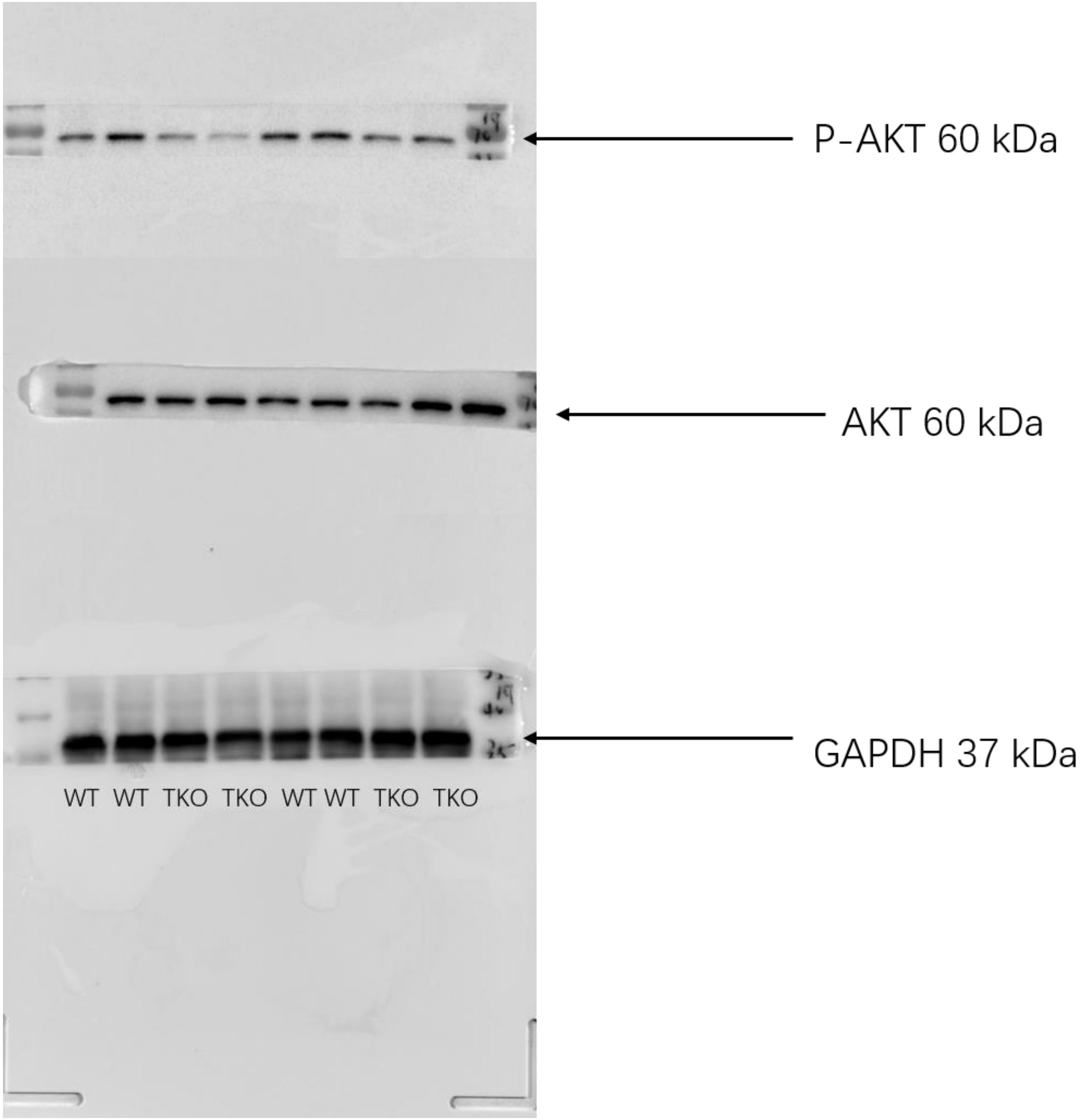

Fig S7. Uncropped blots of Fig 5a.

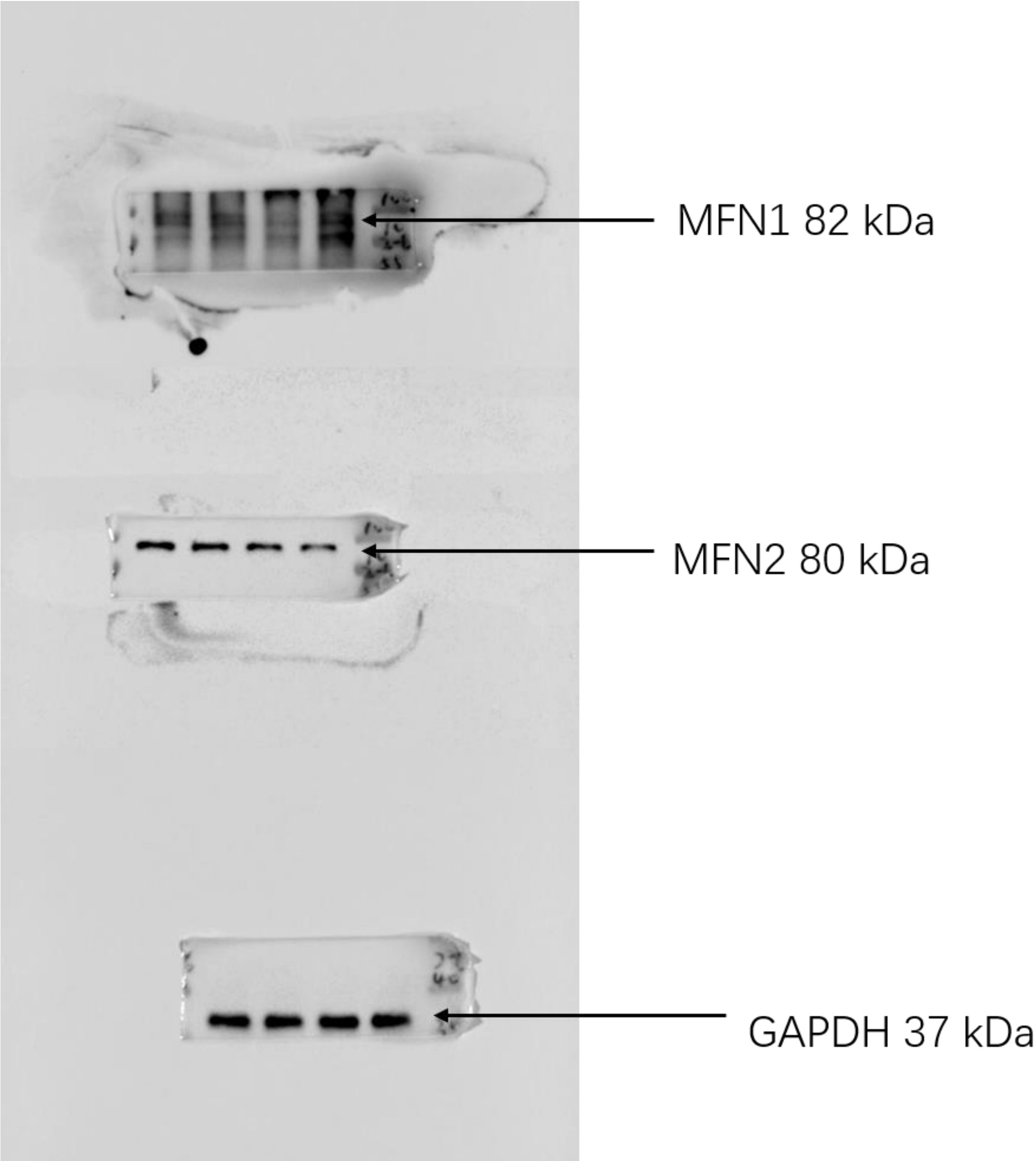

Fig S7. Uncropped blots of Fig 5b.

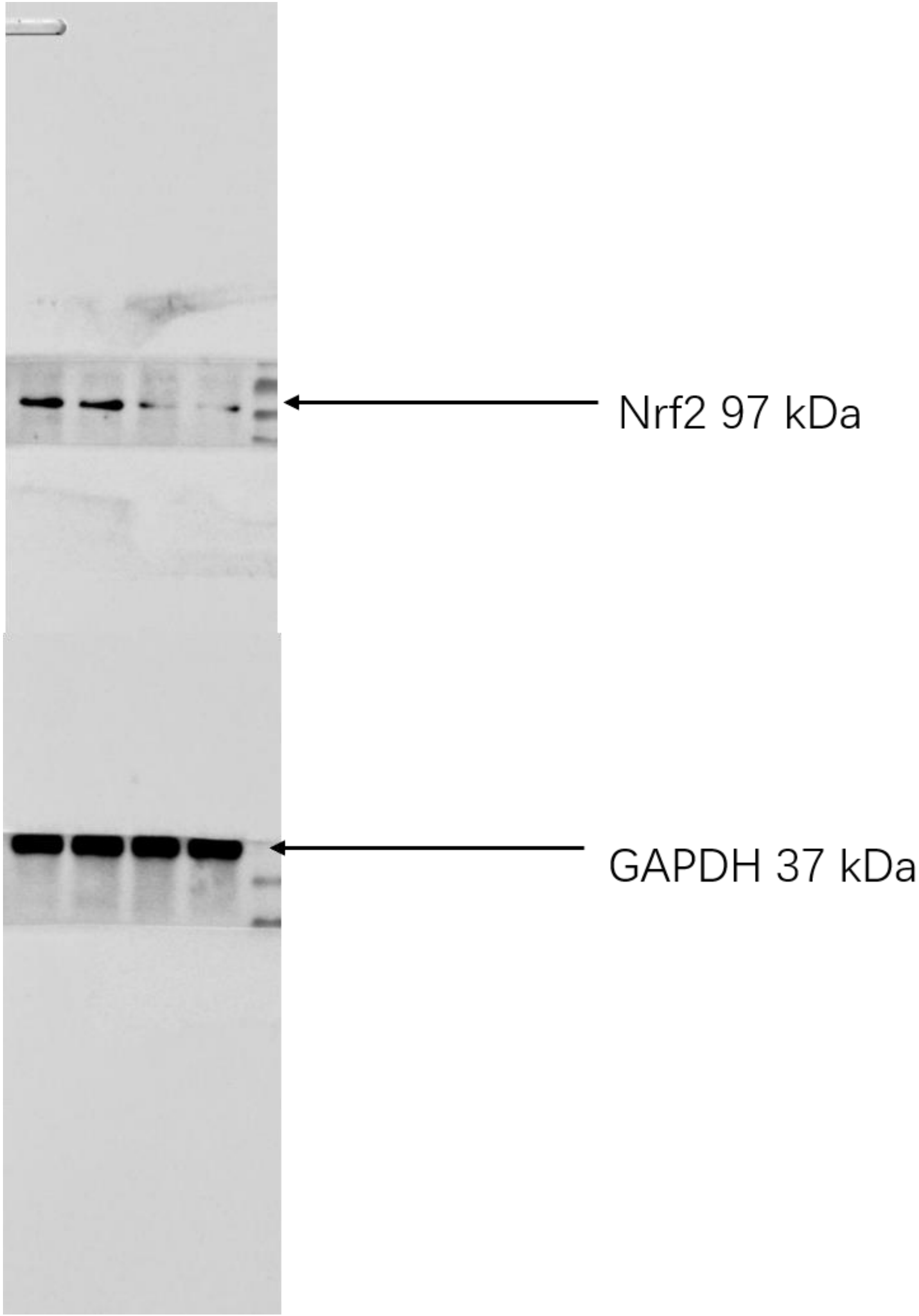

Fig S7. Uncropped blots of Fig 5c.

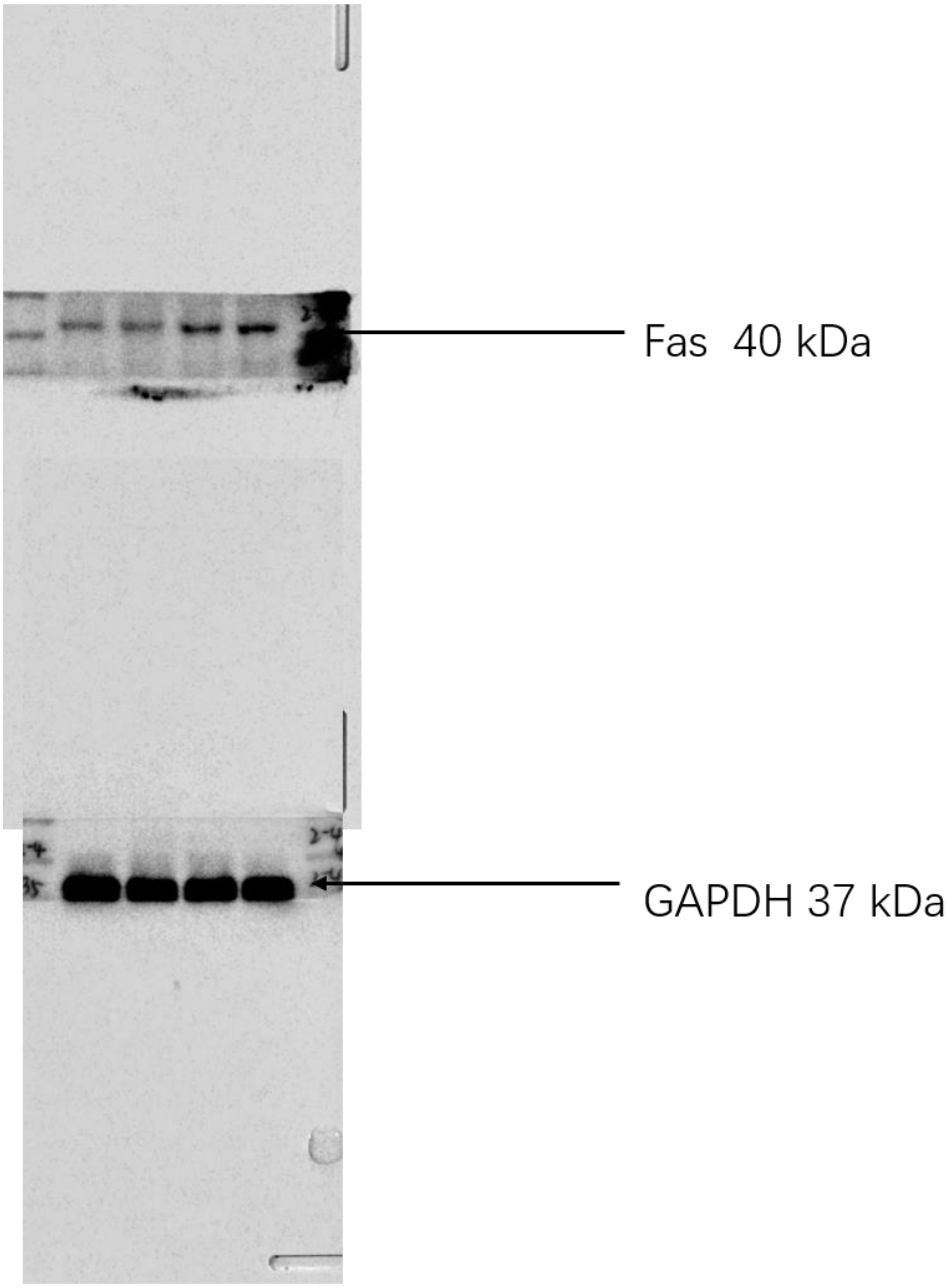

Fig S7. Uncropped blots of Fig 5d.

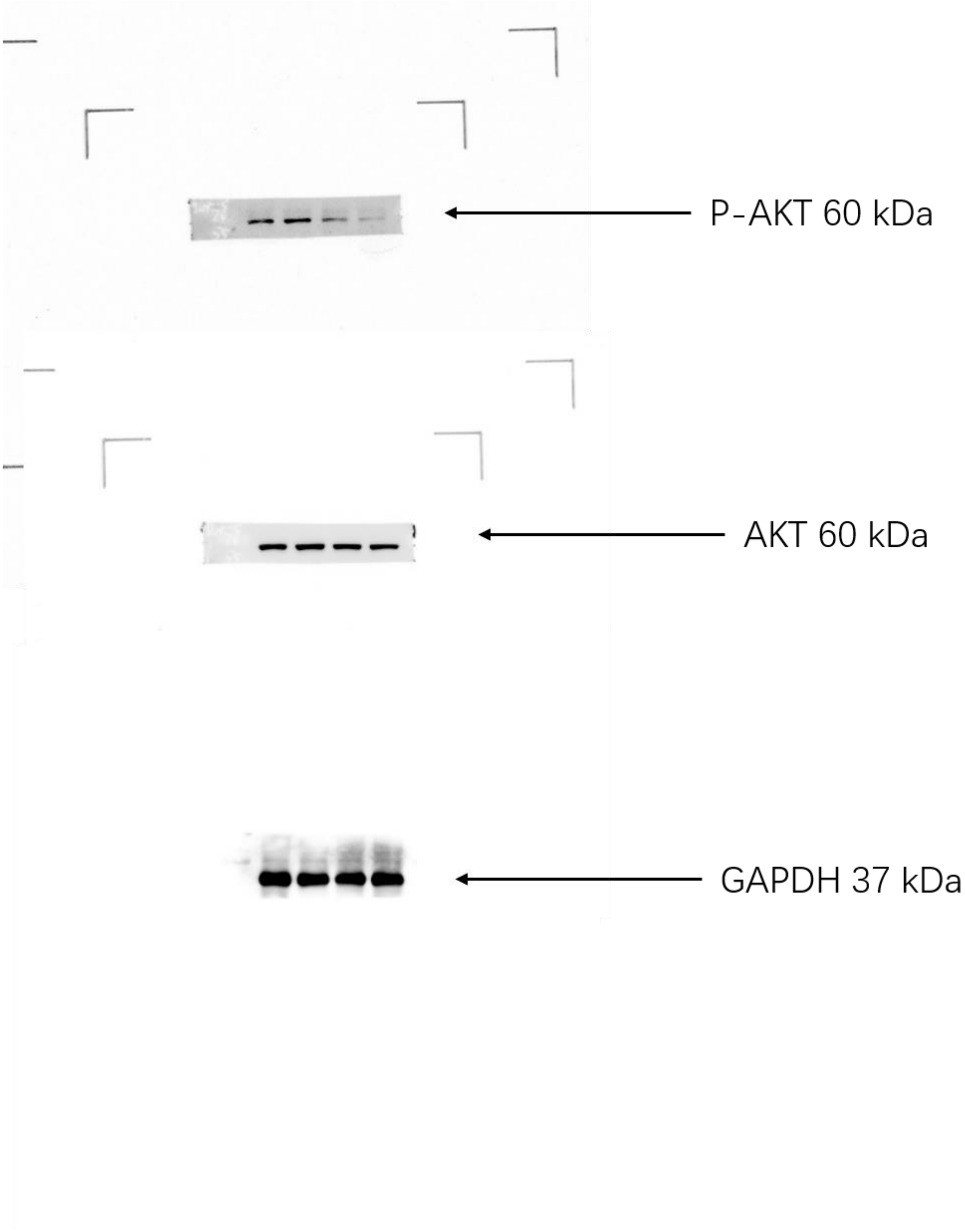

Fig S7. Uncropped blots of Fig 6d.

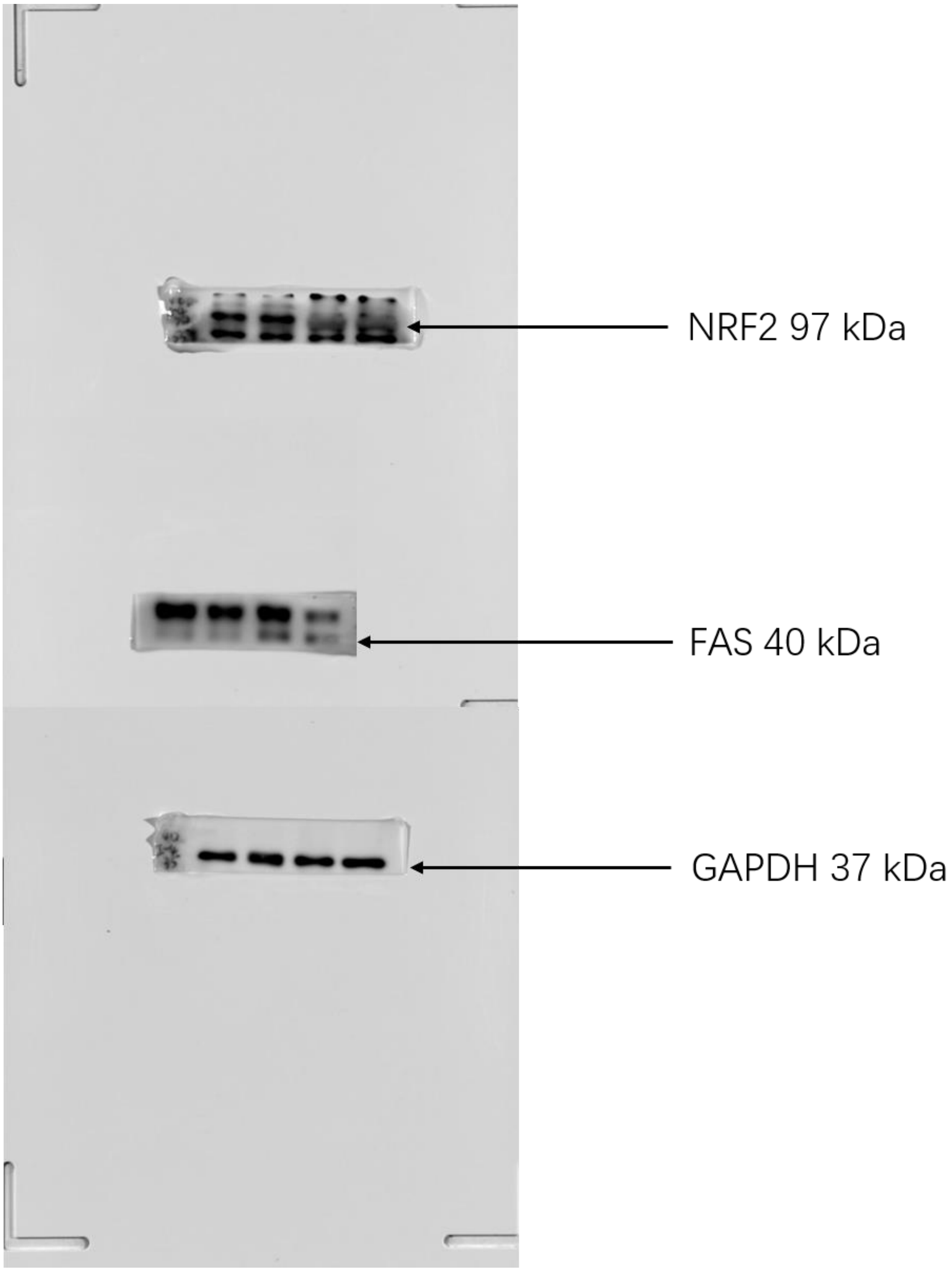

Fig S7. Uncropped blots of Fig 6e.

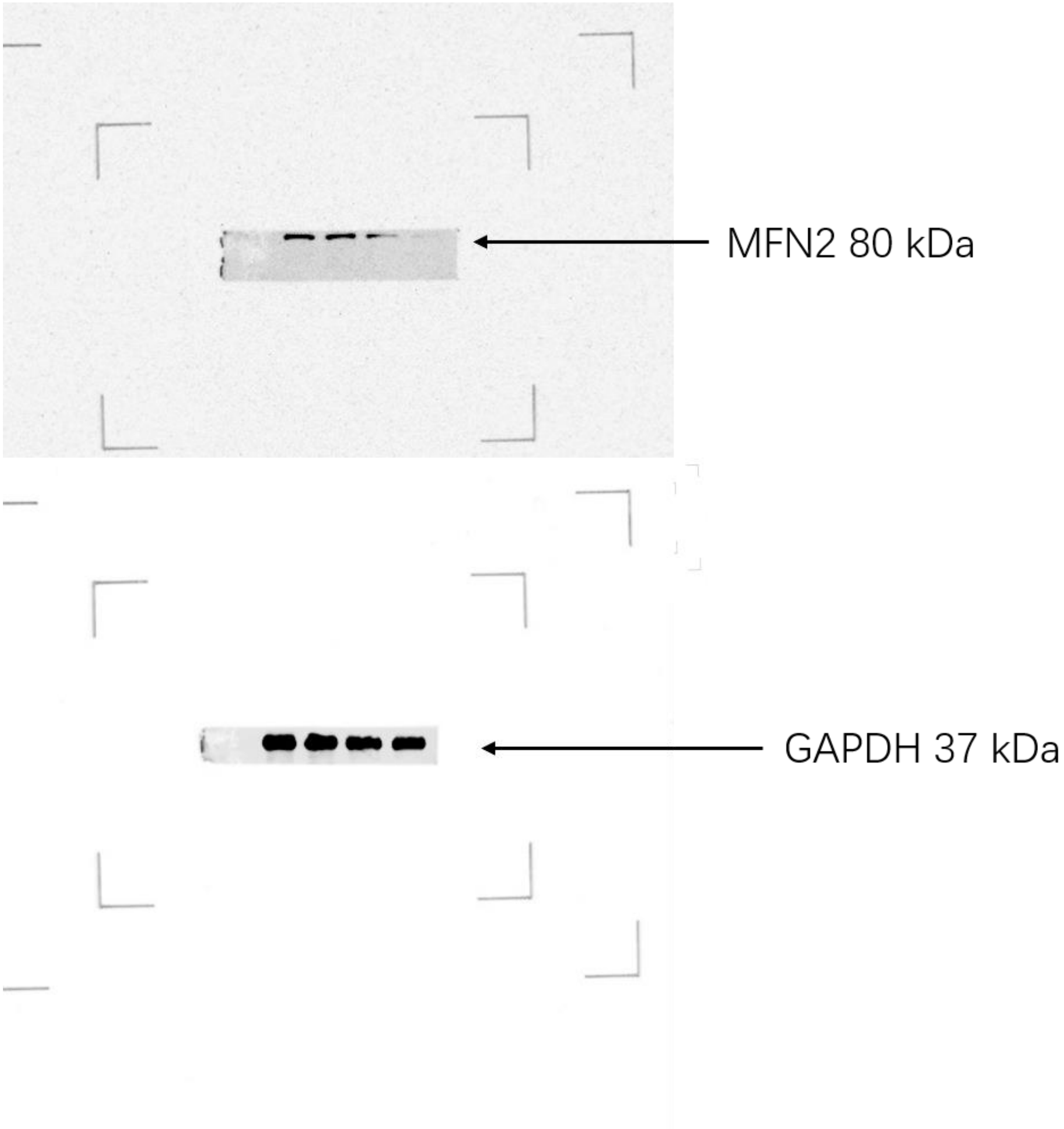

Fig S7. Uncropped blots of Fig 6f.

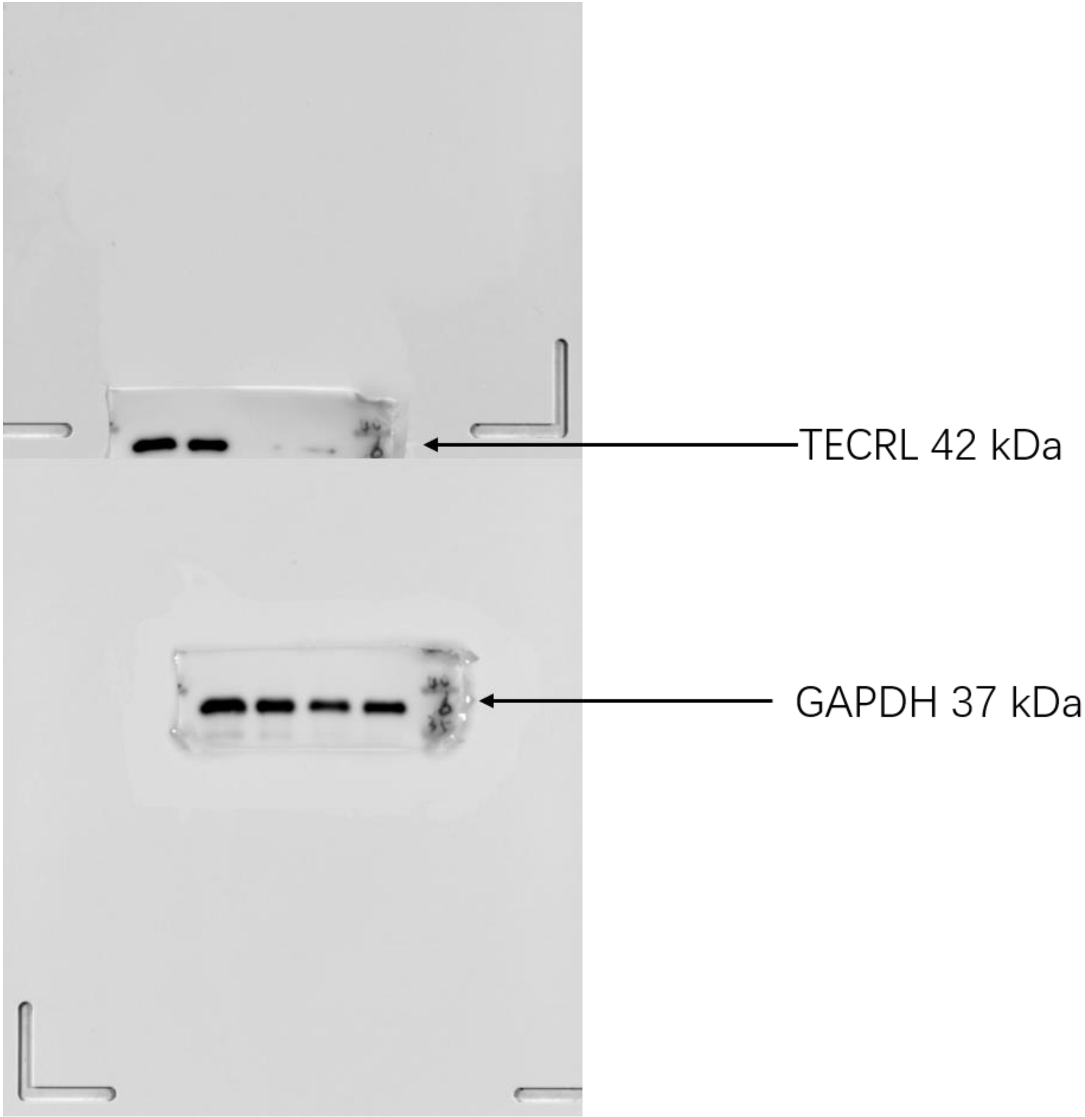

Fig S7. Uncropped blots of Fig 6g.

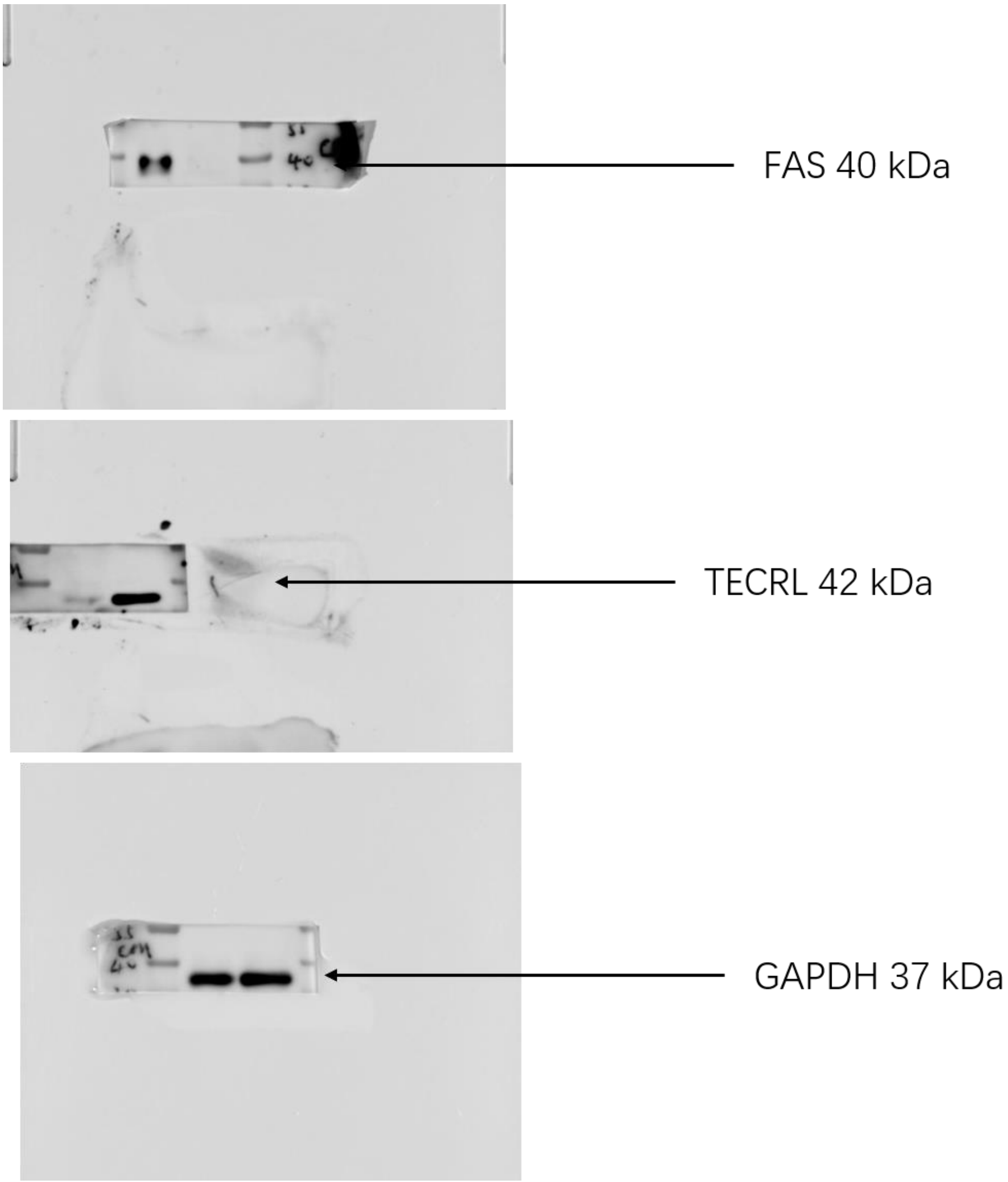

Fig S7. Uncropped blots of Fig 7a.

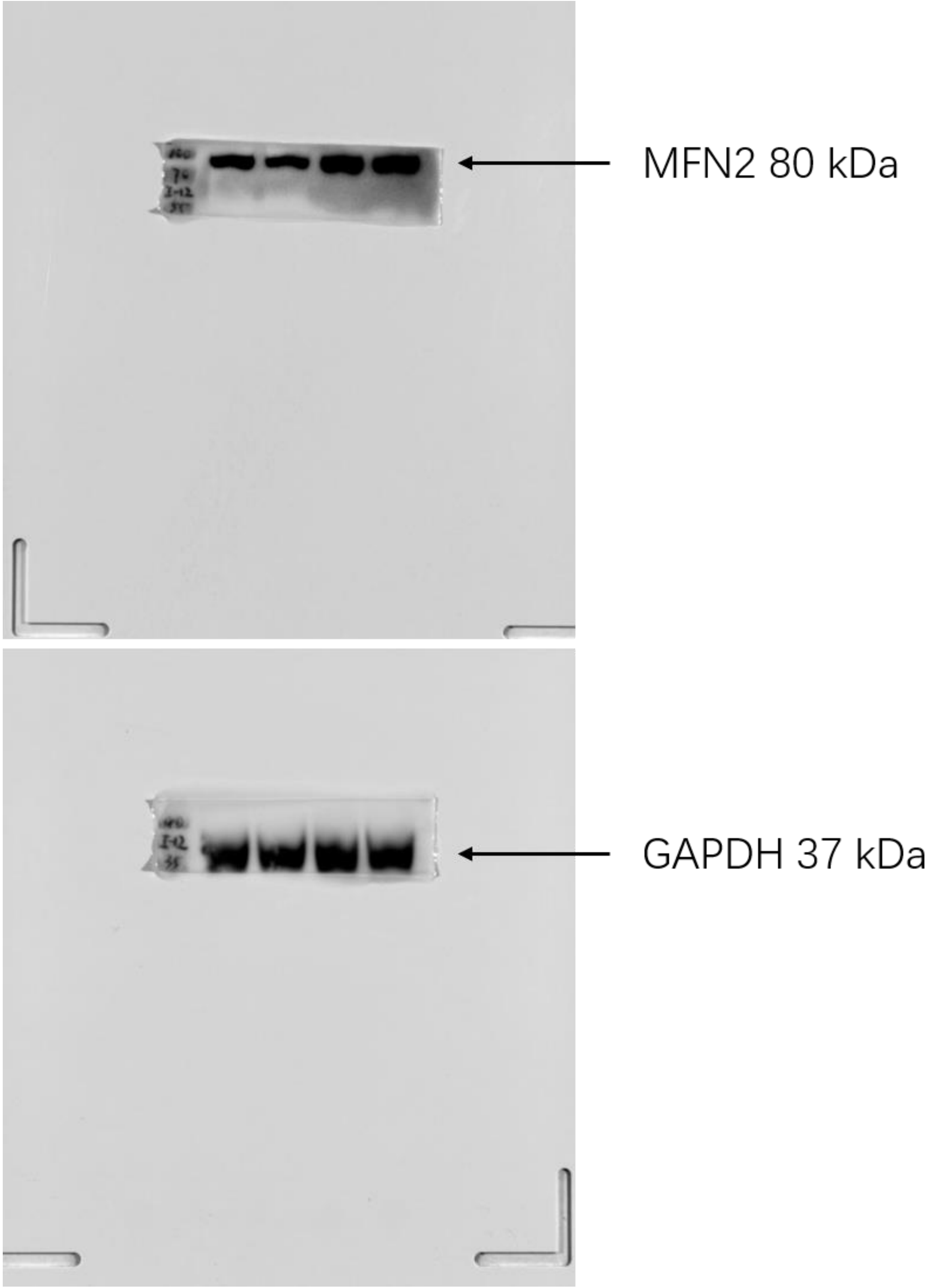

Fig S7. Uncropped blots of Fig 7b.

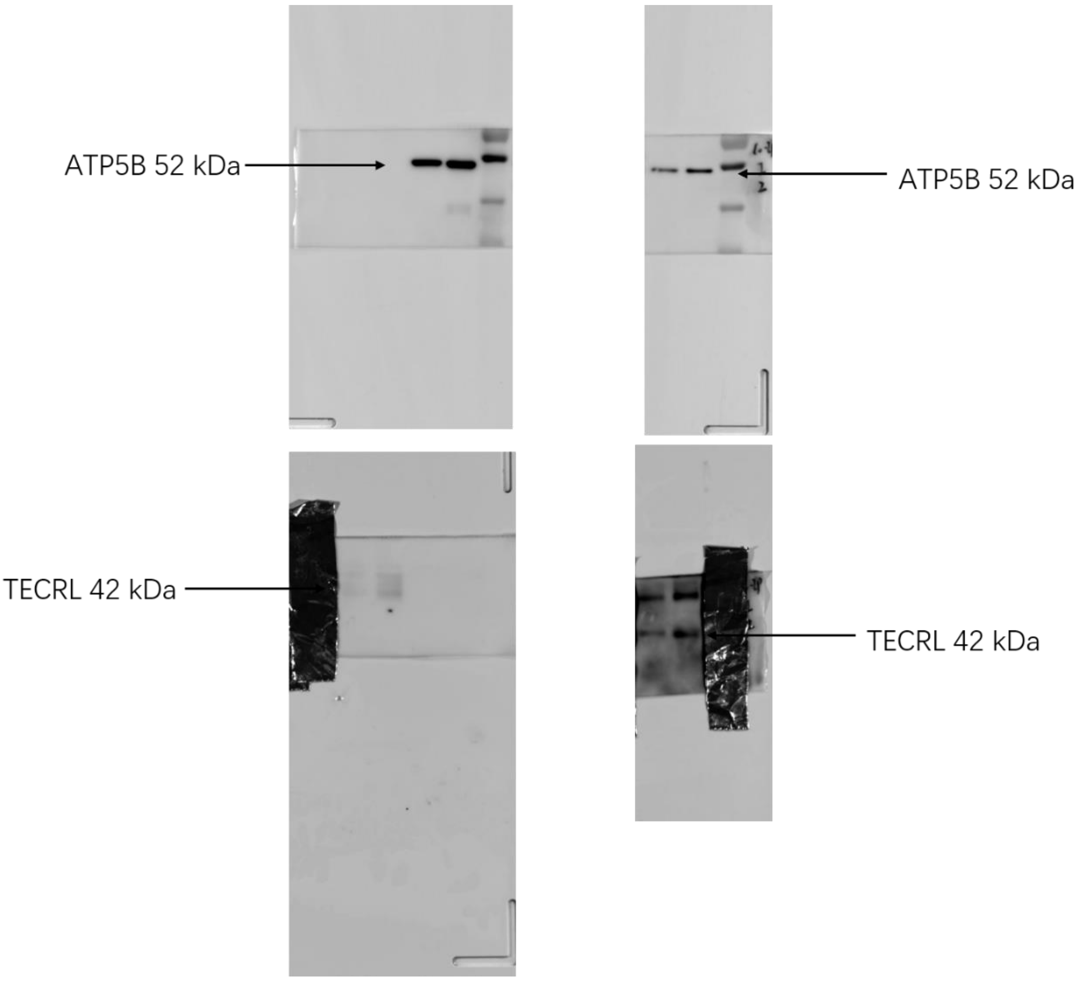

Fig S7. Uncropped blots of Fig 7c.

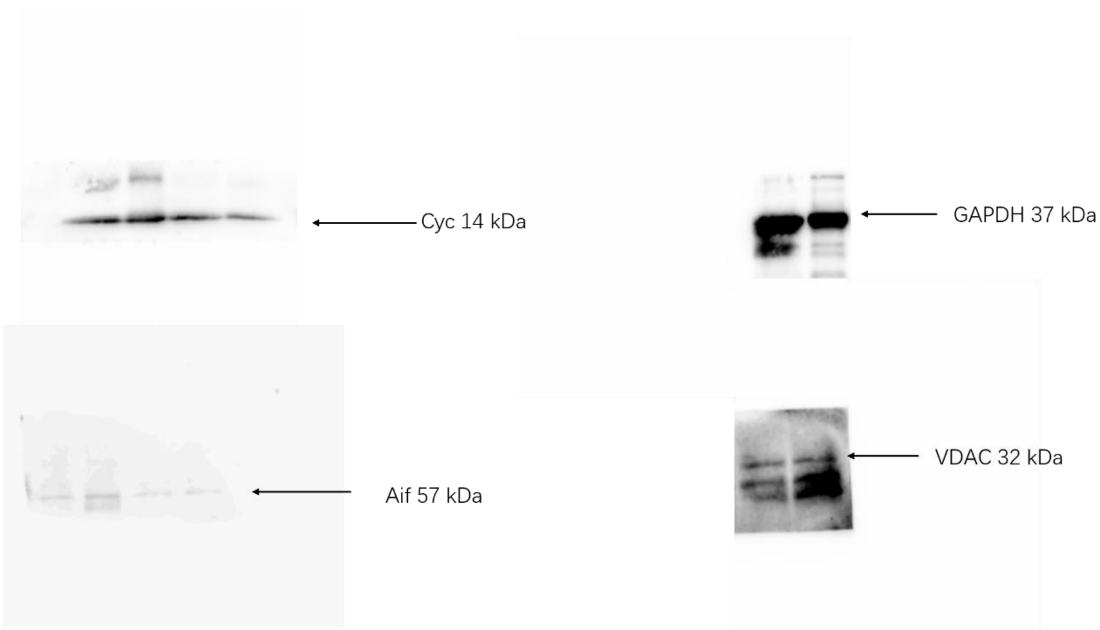

Fig S7. Uncropped blots of Fig 7g.

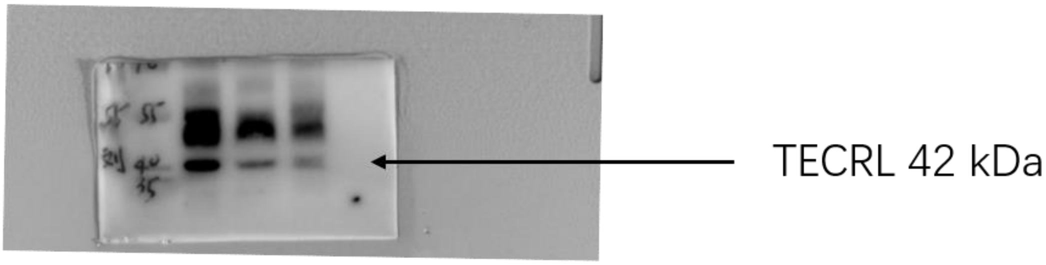

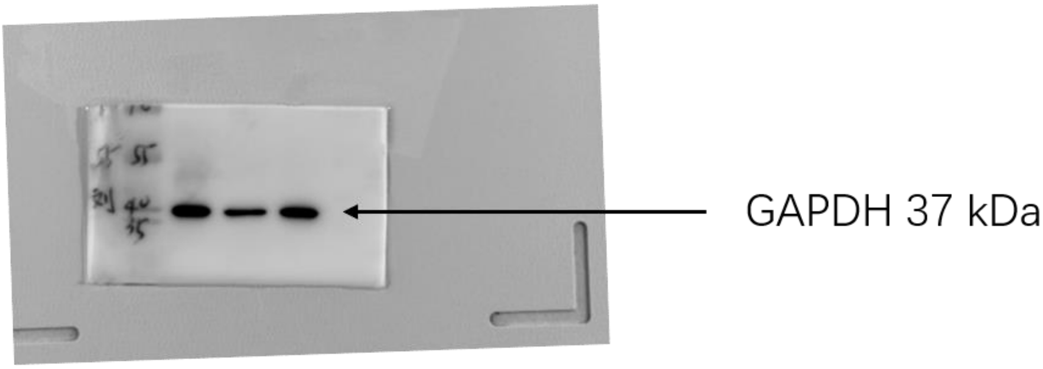

Fig S7. Uncropped blots of Fig s1c.

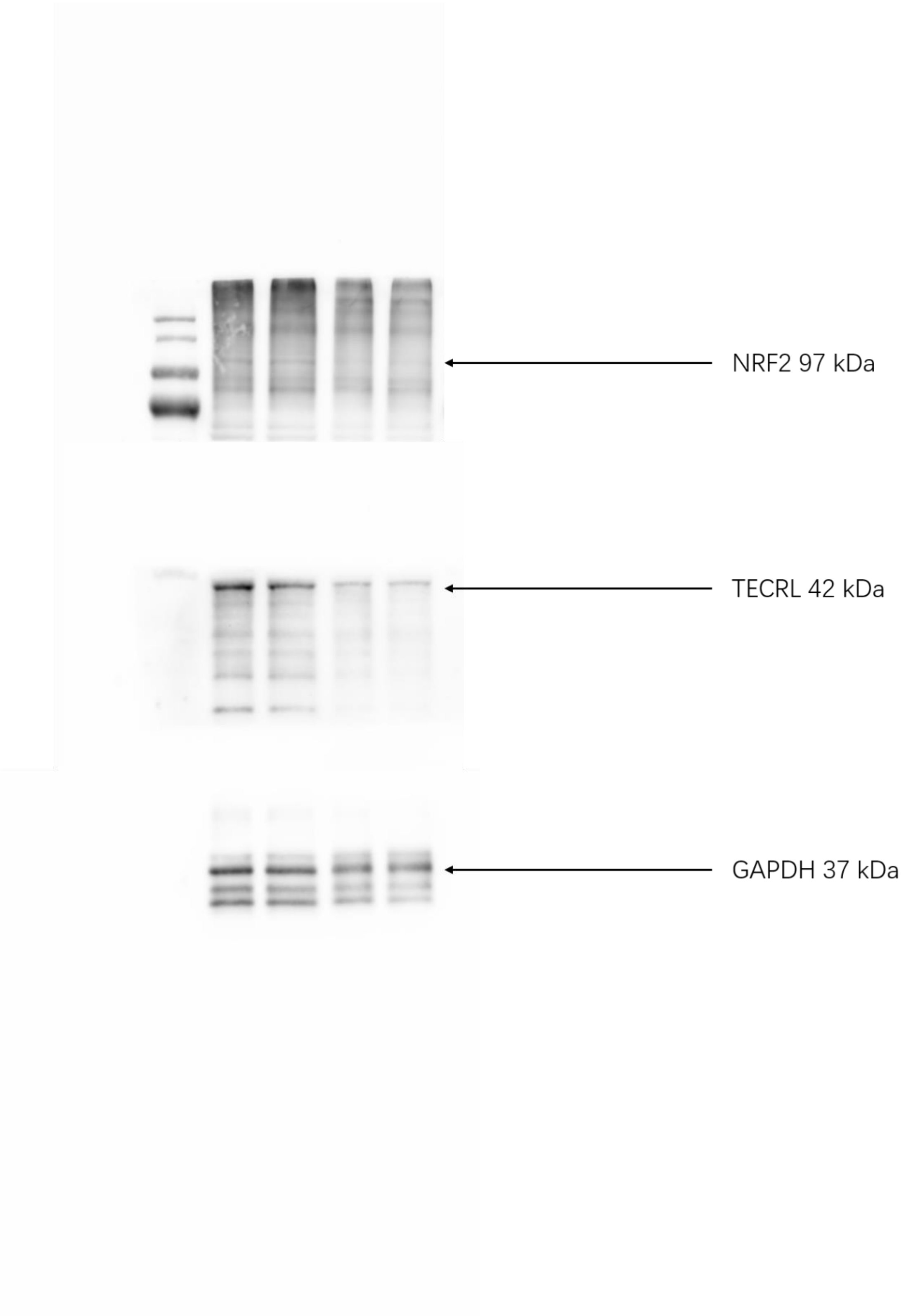

Fig S7. Uncropped blots of Fig s5a.

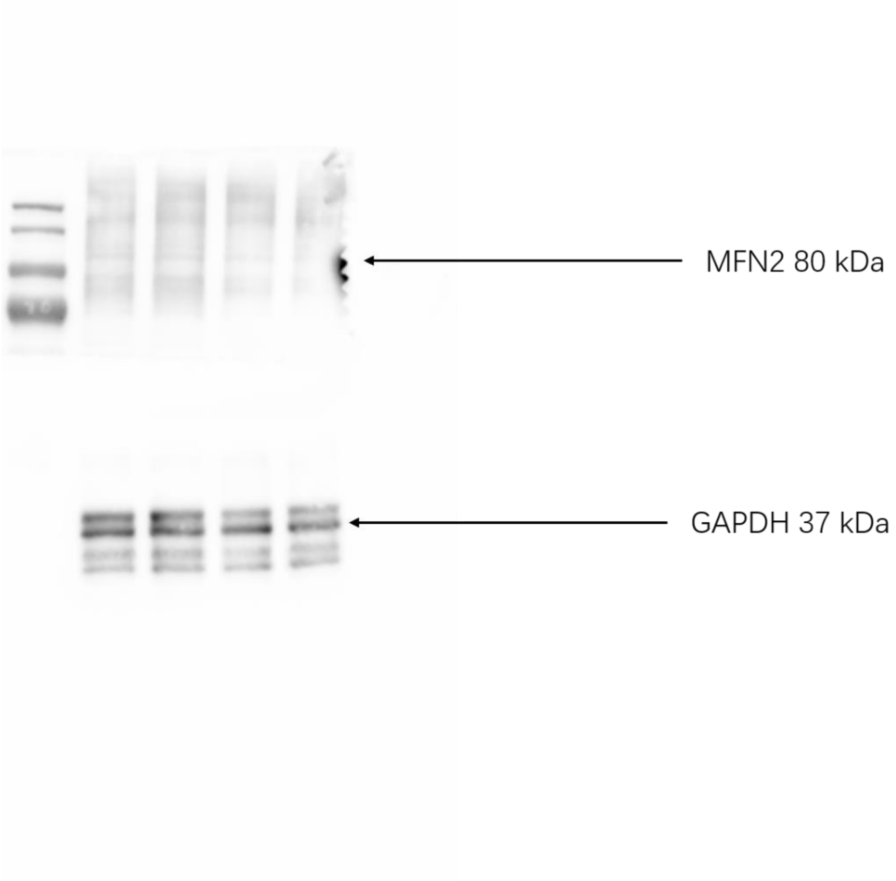

Fig S7. Uncropped blots of Fig s5b.

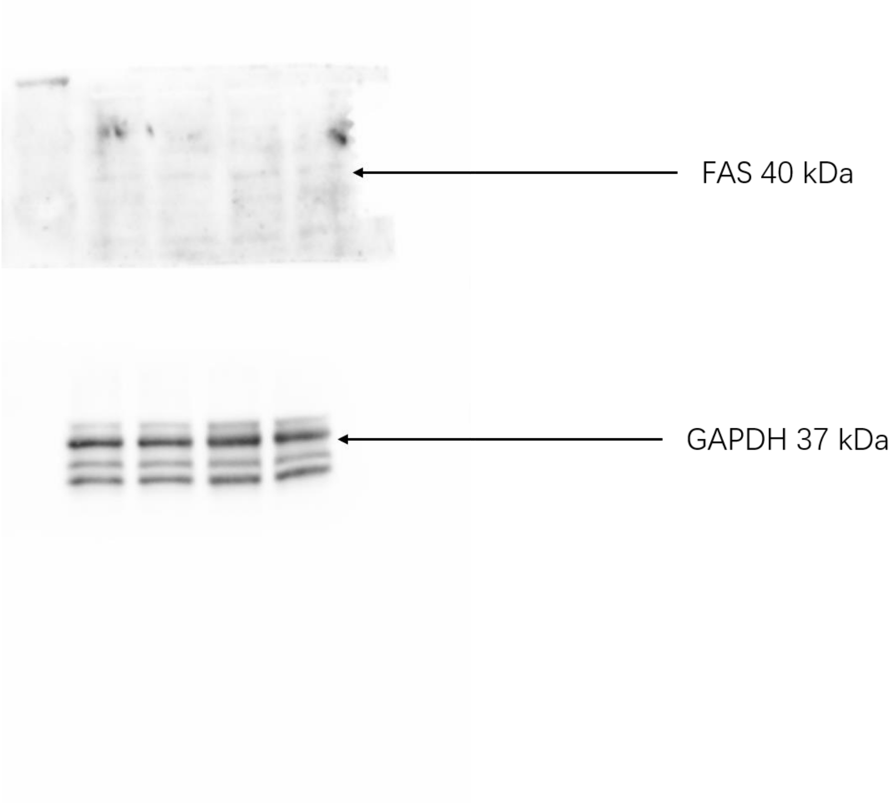

Fig S7. Uncropped blots of Fig s5c.
