# Supplementary material for: TECRL deficiency results in aberrant mitochondrial function in cardiomyocytes
Source: Commun Biol. 2022 May 16;5:470. doi: 10.1038/s42003-022-03414-9 (PMC9110732; doi:10.1038/s42003-022-03414-9)
Supplement: Supplementary file 3 — Reporting Summary [file 42003_2022_3414_MOESM3_ESM.pdf]

nature portfolio

Corresponding author(s): Tingting Xiao

Last updated by author(s): Mar 23, 2022

Reporting Summary

Nature Portfolio wishes to improve the reproducibility of the work that we publish. This form provides structure for consistency and transparency in reporting. For further information on Nature Portfolio policies, see our Editorial Policies and the Editorial Policy Checklist.

Statistics

For all statistical analyses, confirm that the following items are present in the figure legend, table legend, main text, or Methods section.

n/a	Confirmed	
☐	☒	The exact sample size (n) for each experimental group/condition, given as a discrete number and unit of measurement
☐	☒	A statement on whether measurements were taken from distinct samples or whether the same sample was measured repeatedly
☐	☒	The statistical test(s) used AND whether they are one- or two-sided<br>*Only common tests should be described solely by name; describe more complex techniques in the Methods section.*
☐	☒	A description of all covariates tested
☐	☒	A description of any assumptions or corrections, such as tests of normality and adjustment for multiple comparisons
☐	☒	A full description of the statistical parameters including central tendency (e.g. means) or other basic estimates (e.g. regression coefficient) AND variation (e.g. standard deviation) or associated estimates of uncertainty (e.g. confidence intervals)
☐	☒	For null hypothesis testing, the test statistic (e.g. F, t, r) with confidence intervals, effect sizes, degrees of freedom and P value noted<br>*Give P values as exact values whenever suitable.*
☒	☐	For Bayesian analysis, information on the choice of priors and Markov chain Monte Carlo settings
☒	☐	For hierarchical and complex designs, identification of the appropriate level for tests and full reporting of outcomes
☒	☐	Estimates of effect sizes (e.g. Cohen's d, Pearson's r), indicating how they were calculated

Our web collection on statistics for biologists contains articles on many of the points above.

Software and code

Policy information about availability of computer code

Data collection	There is no commercial code in this manuscript.
Data analysis	There is no commercial code in this manuscript.

For manuscripts utilizing custom algorithms or software that are central to the research but not yet described in published literature, software must be made available to editors and reviewers. We strongly encourage code deposition in a community repository (e.g. GitHub). See the Nature Portfolio guidelines for submitting code & software for further information.

Data

Policy information about availability of data

All manuscripts must include a data availability statement. This statement should provide the following information, where applicable:

- Accession codes, unique identifiers, or web links for publicly available datasets
- A description of any restrictions on data availability
- For clinical datasets or third party data, please ensure that the statement adheres to our policy

All relevant data are available by request from the authors.

Field-specific reporting

Please select the one below that is the best fit for your research. If you are not sure, read the appropriate sections before making your selection.

☒ Life sciences ☐ Behavioural & social sciences ☐ Ecological, evolutionary & environmental sciences

For a reference copy of the document with all sections, see nature.com/documents/nr-reporting-summary-flat.pdf

Life sciences study design

All studies must disclose on these points even when the disclosure is negative.

Sample size	Most of the experiments in the manuscript are more than six.
Data exclusions	According to the statistical method, the outliers in the data were removed.
Replication	Most of the experiments in the manuscript were repeated six times.
Randomization	Animal experiments were repeated with more than 6 mice. For cell experiments, 6 independent cells were used to replicate.
Blinding	Information on the frequency and duration of arrhythmias was double-blind recorded and analyzed by skilled clinicians.

Reporting for specific materials, systems and methods

We require information from authors about some types of materials, experimental systems and methods used in many studies. Here, indicate whether each material, system or method listed is relevant to your study. If you are not sure if a list item applies to your research, read the appropriate section before selecting a response.

Materials & experimental systems

n/a	Involved in the study	
☐	☒	Antibodies
☐	☒	Eukaryotic cell lines
☒	☐	Palaeontology and archaeology
☐	☒	Animals and other organisms
☒	☐	Human research participants
☒	☐	Clinical data
☒	☐	Dual use research of concern

Methods

n/a	Involved in the study	
☒	☐	ChIP-seq
☒	☐	Flow cytometry
☒	☐	MRI-based neuroimaging

Antibodies

Antibodies used	OCT4 antibody (Cell Signaling Technology, USA), or α-actinin antibody (Cell Signaling Technology, USA), Tecrl (Aviva Systems Biology, USA), Mfn1, Mfn2, Nrf2, Akt, p-Akt (Ser473), Fas, and GAPDH (Cell Signaling Technology, USA).
Validation	All of the antibodies were confirmed by the manufacturer's websites.

Eukaryotic cell lines

Policy information about cell lines

Cell line source(s)	H9C2
Authentication	H9C2 cells were puchased from Shanghai Cell Bank, Chinese Academy of Sciences, China.
Mycoplasma contamination	We confirm that all cell lines in the manuscript are negative.
Commonly misidentified lines (See ICLAC register)	No common cell lines were misdiagnosed in this study.

Animals and other organisms

Policy information about studies involving animals; ARRIVE guidelines recommended for reporting animal research

Laboratory animals	C57BL6, male, 4-5 weeks and 8 weeks.

Wild animals	WT and Tecrl KO mice were used for all experiments at the age of four to five and eight weeks old. Mice were anaesthetized with isoflurane (1%) and moved to a workbench.
Field-collected samples	Animals were raised under controlled conditions (12 h dark-light cycle, 22±2?, and 45–55% relative humidity) and housed with a maximum of five animals per cage, with unrestricted access to food and water.
Ethics oversight	All animal studies were approved by the Institutional Review Board of Shanghai Children's Hospital, Shanghai Jiaotong University.

Note that full information on the approval of the study protocol must also be provided in the manuscript.
